# Influence of Patella Position on Soft Tissue Balance and Clinical Outcomes in Patients Undergoing Minimally Invasive Total Knee Arthroplasty, a Randomized Clinical Trial

**DOI:** 10.3389/fsurg.2022.692072

**Published:** 2022-02-04

**Authors:** Kaiyuan Liu, Yuxin Liao, Dong Yang, Tianyang Xu, Qiuming Gao, Wenwei Jiang, Lin Fan, Pengfei Zan, Guodong Li

**Affiliations:** ^1^Department of Orthopedics, Shanghai Tenth People's Hospital, Tongji University School of Medicine, Shanghai, China; ^2^Department of Orthopedics, Shanghai First People's Hospital, Jiaotong University School of Medicine, Shanghai, China

**Keywords:** patellar position, MIS-TKA, gap balance, soft tissue balance, clinical effectiveness

## Abstract

**Background:**

We hypothesized that subluxating patellar during minimally invasive total knee arthroplasty (MIS-TKA) would affect intraoperative soft tissue balance and postoperative clinical outcome.

**Methods:**

From December 2018 to May 2020, 189 patients receiving primary MIS-TKA were enrolled. The gap-balance technique was used, with patients randomly assigned to undergo osteotomy and balance of soft tissue with patella reduced (group A; *n* = 93) or subluxated (group B; *n* = 96). The gap and varus?valgus angle were compared between groups in both extension and flexion position. The gap and varus?valgus angle were also compared before and after reducing patellar in group B. Femoral prosthesis rotation, mechanical femoral axis–to–tibial axis angle, Knee Society Score (KSS), visual analog scale (VAS), and range of motion (ROM) were compared postoperatively between two groups. Follow-up was 12 months.

**Results:**

The flexion gap and the varus angle were significantly greater (0.4 mm and 0.7 degree) after patella reduction than before reduction, but the extension joint gap and varus angle were comparable before and after patella reduction. The femoral prosthesis tended to be internally rotated (0.65 degree) in group B. ROM and VAS was better in the group A than in group B at 1 month after surgery, but the differences were not significant at 3, 6 and 12 months. KSS was comparable between the groups after surgery.

**Conclusions:**

During MIS-TKA, as far as possible, soft tissue balance should be achieved with the patella reduced; otherwise, the femoral prosthesis may be installed more internally and, after patella reduction, the flexion gap and varus angle would increase.

**Clinical Trial Registration:**

Current Controlled Trials ChiCTR2000034106, https://www.chictr.org.cn/hvshowproject.aspx?id=39987.

## Introduction

Total knee arthroplasty (TKA) is widely used to treat end-stage knee arthritis (1, 2). Success of surgery depends on accurate osteotomy, accurate reconstruction of lower limb alignment, and achievement of the correct soft tissue balance. While the accuracy of osteotomies and prostheses placement has improved vastly with the introduction of computer navigation and three-dimensional (3D) printed prostheses, achieving soft tissue balance still depends on the surgeon's subjective assessment (3, 4); too much soft tissue laxity can lead to joint instability and possibly even revision surgery, whereas excessive tightness will interfere with postoperative functional training and lead to dysfunction and reduced life of the prosthesis (5–7).

For optimum soft tissue balance, the surgeon must obtain the same extension and flexion gaps and the same medial and lateral gaps (8). Usually, these balances are obtained without reduction of the patella. However, more and more surgeons are stressing the need to assess soft tissue balance under physiological conditions, that is, with the patella reduced. In conventional TKA, the patella is everted during the operation; this eversion affects soft tissue balance, but exactly how much impact it has is still debated (9). The effect of patellar eversion on the gap may vary with the type of prosthesis being used—i.e., posterior-cruciate retaining (CR) or posterior-cruciate substituting (PS). The effect on postoperative clinical function is also not yet clear.

Minimally invasive total knee arthroplasty (MIS-TKA) has provided good clinical results (10, 11). During MIS-TKA, the patella is not everted. However, the preservation of the extensor mechanism and the limited exposure actually leads to greater tension on the soft tissues than patellar eversion (12). The lateral dislocation of the patella itself may also has major impact on the soft tissue balance.

The purpose of this study was (1) to determine the effect of different patella positions on soft tissue balance during MIS-TKA. (2) to find whether the femoral component would be implanted internally or externally when the gap technique was performed with the patellar shifted laterally in MIS-TKA and (3) to evaluate the difference in clinical effectiveness of TKA between patients achieving soft tissue balance with the patella in different positions.

## Methods

### Patients

From among the 254 patients who underwent MIS-TKA at our center between December 2018 and May 2020, we recruited 189 patients for this randomized clinical trial in Shanghai Tenth People's Hospital. Patients were eligible for inclusion if they (1) had osteoarthritis, with Kellgren–Lawrence (K-L) grade 3–4; (2) were undergoing primary TKA; and (3) had varus or neutral knee. Patients were excluded if they had (1) rheumatoid arthritis, (2) history of surgery or infection of the knee, (3) ligament injury of the knee, (4) valgus deformity, or (5) severe bone defects that might require insertion of augments. A total of 65/254 patients were excluded: 39 patients with K-L grade 2 severity, 13 with rheumatoid arthritis, 8 with severe varus knees and obvious bone defect, and 5 with valgus deformity. The remaining 189 patients were randomly separated into two groups using a completely randomized digital table: one group was assigned to receive patellar reduction (group A; *n* = 93) and the other to receive patellar subluxation (group B; *n* = 96) during surgery. A colleague from our outpatient department helped enroll participants and assign them to different groups.

This study was approved by the hospital ethics committee and it follows the WMA Declaration of Helsinki 2013 for medical research involving human subjects. And written informed consent was obtained from each patient. Operations were performed by the same senior surgeon. The patients and the staff who assessed postoperative efficacy were blinded to the intervention.

### General Preoperative and Postoperative Measures

Anteroposterior long-leg standing radiograph was taken to guide the selection of the valgus angle of the femoral component. Computed tomography (CT) scan of the knee was also acquired preoperatively. An antibiotic (second-generation cephalosporin) was routinely administered intravenously 20 min before surgery. The postoperative protocol for pain relief was the same for all patients. There was no blood drains after surgery. The long-leg standing radiograph and CT of the operated knee were repeated at 5–7 days after operation. All patients received the same postoperative rehabilitation.

### Surgical Technique

A gap-balance technique was used in the operation (13). All patients received a PS prosthesis (GENESIS II; Smith & Nephew, USA). The patient was placed in the supine position, and an air tourniquet applied at 250 mm Hg; the operation was performed under tourniquet control and the tourniquet was loosened immediately after prosthesis implantation. Midline skin incision and the midvastus approach were used to expose the knee without everting the patella. The anterior cruciate ligament and meniscus were excised, and the distal femur was exposed. In our study, the surgeon would balance the extension gap prior to the flexion gap. According to the preoperative long-leg standing radiograph, the valgus angle of the femoral component (5–7°) was selected. An intramedullary guide was used to perform distal femoral bone cut. After adequate exposure, all osteophytes, posterior cruciate ligament and menisci were removed. Then, the tibia was subluxated anteriorly. The tibia was cut at 90° to the coronal plane of the mechanical axis, using an intramedullary guide (7°of the posterior tibial slope). The posterior inclination along the sagittal plane and the rotational alignment were checked. After the osteotomy, in case of tensioning effect on adjacent ligamentous structures, all osteophytes including posterior femoral ought to be removed at this time before any soft tissue releases. Following extension gap resection, osteophyte removal and soft tissue balance, the gap balance was assessed with a conventional spacer block. The assessment was performed after reduction of the patella in group A patients, and with the patella in the subluxated state in group B patients. In Group B, the patella was subluxated by lateral displacement and no patellar eversion was performed. If there was still asymmetry, tight ligamentous structures such as posterior capsule are further released until a balanced extension gap was obtained in the coronal plane.

**Figure 1:**
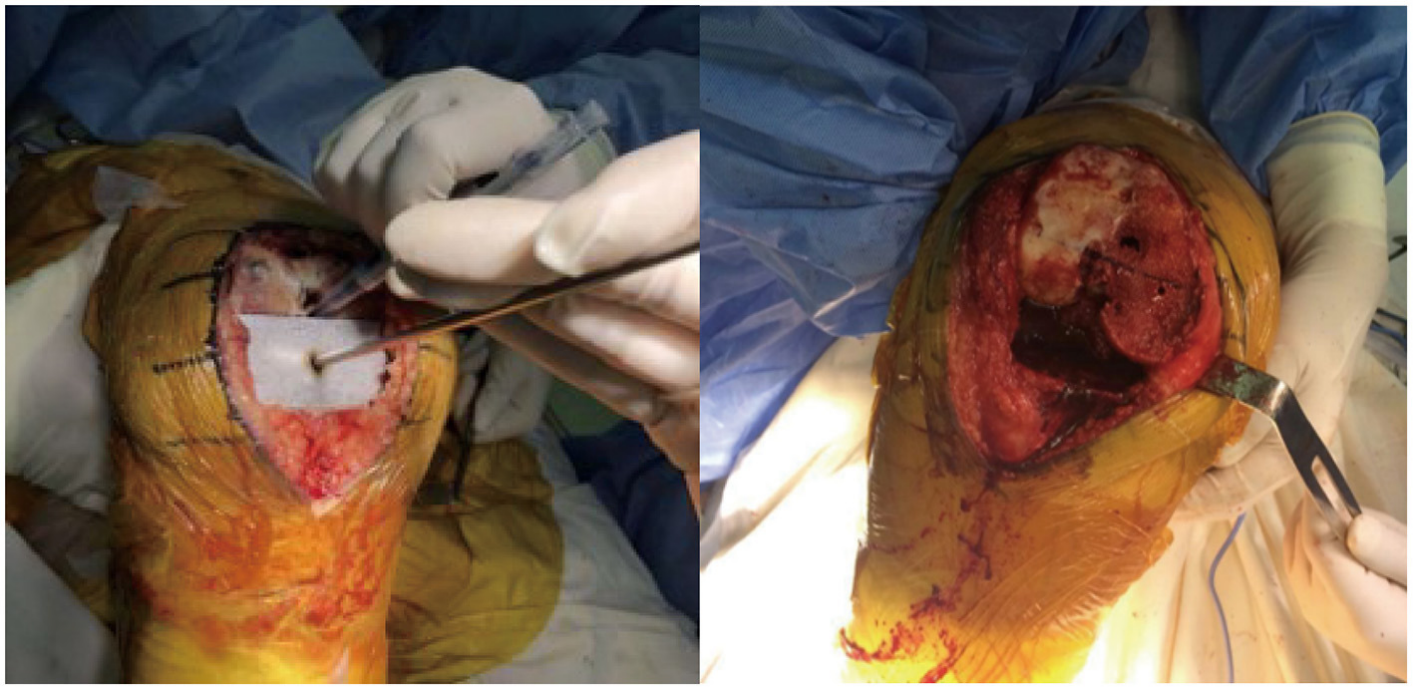
The matching rectangle is used to record the line, and the external osteotomy angle of the femoral posterior condyle is adjusted according to this scribe line with a gap balance technique.

The femoral sizing guide was closely applied to the distal femoral cut surface, and the size of the cutting jig was determined according to the anteroposterior diameter of the femur. A 3.2-mm drill bit was used to drill through the nail hole on the femoral sizing guide, which was then removed. Next, since the operation was performed using a gap balance technique, the external osteotomy angle of the femoral posterior condyle was redetermined by the gravity method: with an assistant lifting the patient's thigh to make the tibia sag naturally. In group A, the patella was maintained in the reduced position while the knee was flexed to 90°. In group B, the patella was first subluxated and then the knee was flexed to 90°. Then the matching rectangle was used to record the line (Figure 1). The positioning hole of the osteotomy module was adjusted according to the scribe line. Then, the anteroposterior cutting block was placed on the distal femoral cut and held in place with three pins. A complete femoral bone cut was performed after confirming that the flexion gap was satisfactory. A femoral trial prosthesis was installed. The matching (left or right) offset-type tensor was selected and fixed to the proximal tibia, and then fitted to the femoral trial prosthesis. The joint distraction force was set at 40 lb in each patient. Gap length and varus ligament imbalance were measured at 0 and 90° of knee flexion. In group A patients the measurements were only made with the patella reduced, whereas in group B patients the measurements were made with the patella laterally shifted and then, again, with the patella reduced.

### Observation Indices

**Figure 2:**
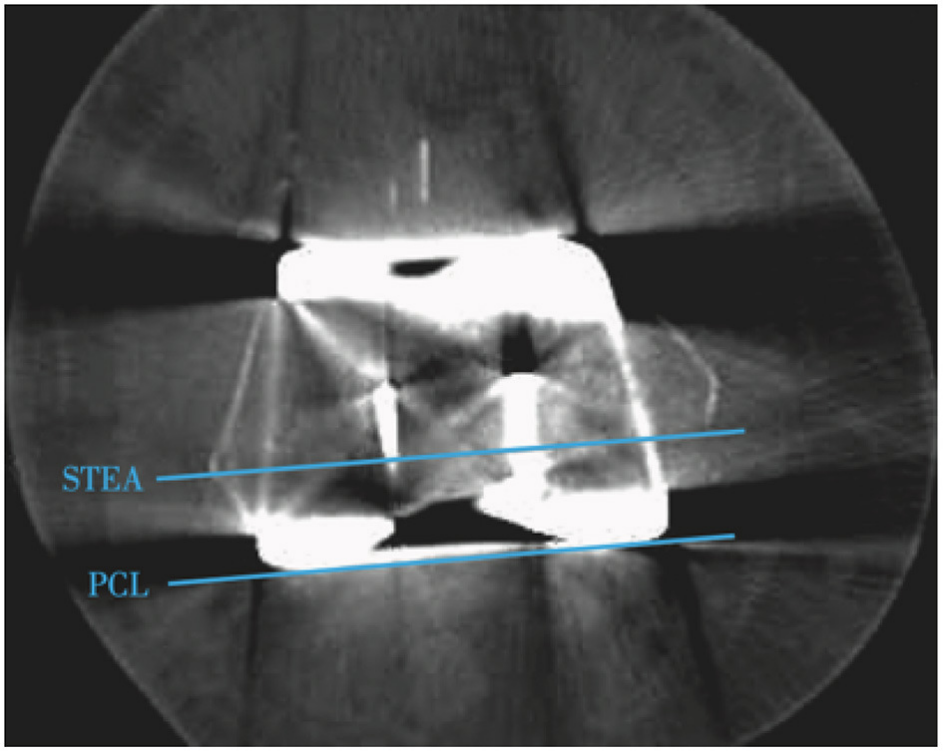
Postoperative CT of knee. The STEA (surgical transepicondylar axis) represents the axis of rotation of the original femur of the knee, while the PCL (posterior condyle line) of the prosthesis can be regarded as the axis of rotation of the prosthesis. The femoral prothesis rotation angle reflects the difference of the femoral prosthesis rotation axis with the surgical transepicondylar axis.

**Figure 3:**
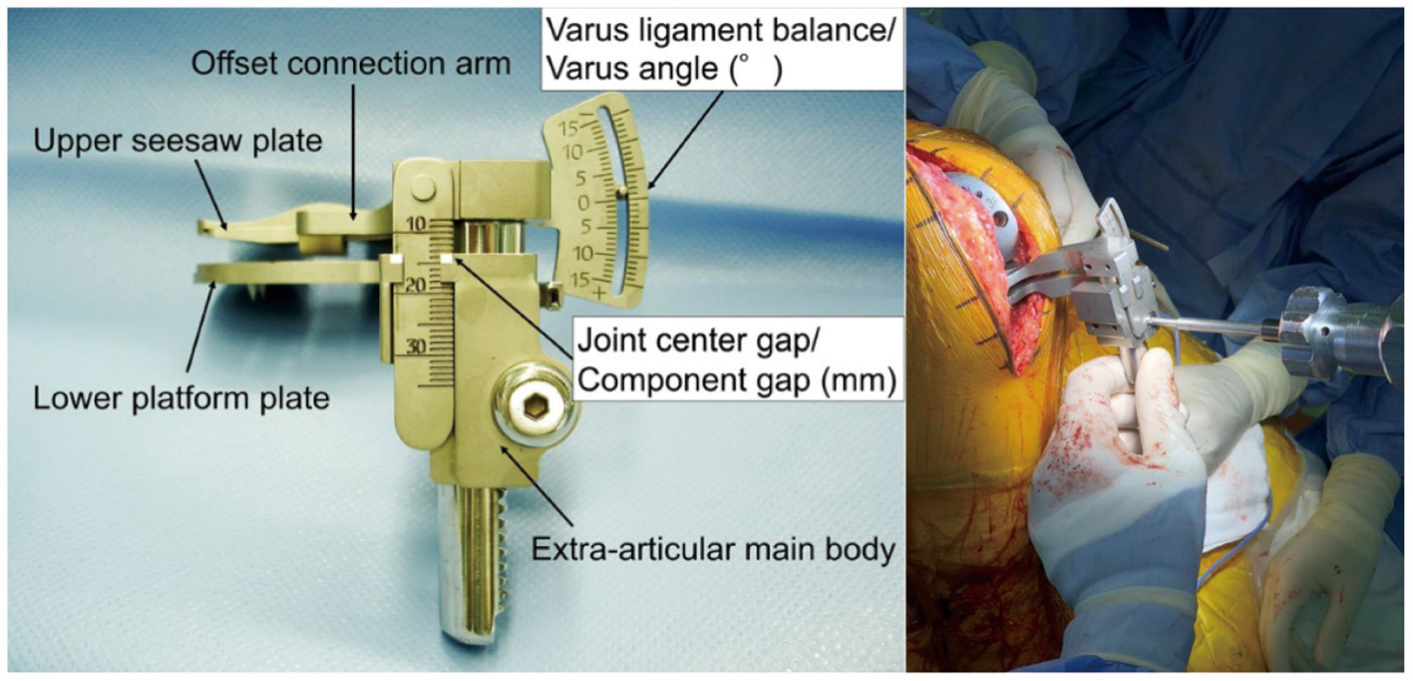
The Offset-Repo-Tensor and the Torque Wrench.

The following were recorded:

1. Basic information (sex, age, height, weight, left or right knee).
2. Femoral rotational alignment and femoral prothesis rotation angle on the CT scan of the knee, the angle between the surgical transepicondylar axis and the posterior condyle line of the femur was the femoral rotational alignment, and the angle between the surgical transepicondylar axis and posterior condyle line of the prosthesis was femoral prosthesis rotation angle (Figure 2). External rotation was indicated with the “plus” sign, and internal rotation with the “minus” sign. The surgical transepicondylar axis represents the axis of rotation of the original femur of the knee joint. The posterior condyle line of the prosthesis can be regarded as the axis of rotation of the prosthesis. The femoral prothesis rotation angle reflects the coincidence of the axis of rotation of the prosthesis with the axis of rotation of the original femur.
3. Mechanical femoral axis–to–tibial axis angle (mFTA).
  The angle between the mechanical axis of the tibia and the mechanical axis of the femur before and after surgery were measured on the long-leg standing radiographs; the target angle after surgery was 0° ± 3°.
4. Offset-Repo-Tensor measurement of joint gap and varus angle (Figure 3).
  As recommended in previous studies (14), the joint gap length and varus angle were measured with the knee in full extension and 90° of flexion. A torque wrench was used to ensure that the joint distraction force was set at 40 lb (Figure 3).
5. Knee Society Score
  KSS score was assessed before surgery and at 1, 3, 6, and 12 months after surgery. The KSS (maximum possible score 100) takes into consideration pain, activity, and stability and reflects knee function.
6. Pain assessment
  A visual analog scale (VAS; ranging from 0 to 10) was used to evaluate knee pain before surgery and at and 1, 3, 6, and 12 months after surgery.
7. Range of motion
  Maximum flexion achievable in the knee was recorded before surgery and at 1, 3, 6, and 12 months after surgery.

### Statistical Analysis

SPSS 25.0 (IBM Corp., Armonk, NY, USA) was used for statistical analysis. Differences between groups in age, body mass index (BMI), femoral rotational alignment, femoral prosthesis rotational alignment, joint gap, varus angle, KSS, and VAS were compared using the independent-samples *t*-test. The joint gap and varus angle in group B before and after reduction were compared using the paired sample *t*-test. Pearson chi-square test was used to compare categorical variables (sex distribution and side affected). Differences in KSS, VAS and ROM between preoperation, 1, 3, 6, and 12 month after operation were compared using one-way analysis of variance. Statistical significance was at *P* ≤ 0.05.

## Result

There were 189 patients who completed the 12 months of follow-up. The two groups were comparable with regard to age, sex distribution, BMI, side affected (left or right), preoperative KSS, preoperative ROM, preoperative VAS, femoral rotational alignment, and preoperative mFTA (*P* > 0.05; Table 1). The operation was uneventful in all patients. Postoperatively, two patients in group A developed deep venous thrombosis (revealed on B-ultrasonography) but improved after anticoagulation; one patient in group B had necrosis of the surgical wound but recovered after 5 weeks of dressing changes.

**Table 1:** Comparison of baseline characteristics between the two groups.

	**Group A** ***n* = 93**	**Group B** ***n* = 96**	***P***
Age, years, mean ± SD (years)	66.8 ± 7.4	68.3 ± 9.1	0.363
Sex (female/male)	78/15	83/13	0.573
BMI, mean ± SD	22.1 ± 1.6	21.5 ± 2.0	0.117
Center/right	45/48	49/47	0.817
KSS, mean ± SD	41.6 ± 10.1	42.1 ± 9.0	0.806
ROM, mean ± SD(°)	102.6°± 9.2°	103.7°± 10.0°	0.580
VAS, mean ± SD	5.0 ± 1.8	5.3 ± 1.6	0.419
Femoral rotational alignment, mean ± SD(°)	3.8 ± 1.2	3.5 ± 1.2	0.289
mFTA, mean ± SD (°)	7.6 ± 2.9	7.3 ± 2.4	0.652

*SD, standard deviation; BMI, body mass index; KSS, Knee Society Score; ROM, range of motion; VAS, visual analog scale; mFTA, mechanical femoral axis–to–tibial axis angle*.

### Intraoperative Soft Tissue Balance

#### Comparison Between Group A and Group B

The soft tissue balance was within a satisfactory range for all patients. There were no significant differences between the groups in joint gap and varus angle in extension or in flexion (*P* > 0.05; Table 2). The group B values here were measured with patellar reduction.

**Table 2:** Comparison of intraoperative balance between the two groups.

**Group**	**Group A** ***n* = 93**	**Group B** ***n* =96**	***P***
Gap at extension(mm)	10.4 ± 0.3	10.4 ± 0.4	0.654
Varus angle at extension(°)	1.5 ± 0.3	1.4 ± 0.3	0.247
Gap at flexion (90°) (mm)	10.7 ± 0.4	10.6 ± 0.3	0.309
Varus angle at flexion (90°) (°)	1.7 ± 0.5	1.6 ± 0.3	0.096

*The group B values here were measured with patellar reduction. Values are the means ± standard deviation*.

#### Comparison Before and After Patellar Reduction in Group B

In 90° of flexion, the joint gap was 11.0 ± 0.4 mm after patella reduction vs. 10.6 ± 0.3 mm (P < 0.01) before patella reduction, and the gap varus angle was 2.3° ± 0.4° after reduction vs. 1.6° ± 0.3° before reduction (*P* < 0.01). With the knee extended, the differences in joint gap and varus angle before and after patella reduction were not statistically significant (*P* > 0.05; Table 3).

**Table 3:** Comparison of balance before and after reduction of patella in group B.

**Position of patellar**	**Subluxation**	**Reduction**	***P***
Gap at extension(mm)	10.4 ± 0.4	10.4 ± 0.3	0.253
Varus angle at extension(°)	1.4 ± 0.3	1.5 ± 0.4	0.188
Gap at flexion (90°) (mm)	10.6 ± 0.3	11.0 ± 0.4	<0.001^*^
Varus angle at flexion (90°) (°)	1.6 ± 0.3	2.3 ± 0.4	<0.001^*^

*Values are the means ± standard deviation*.

* *means there is significant difference between groups*.

### Postoperative Evaluation

#### Imaging Evaluation

The rotational angle of the femoral component was 0.12° ± 1.3° in group A and more than −0.53° ± 1.3° in group B; the difference was statistically significant (P = 0.005). The angle of the lower limb mechanical axis was corrected to within 0° ± 3° in both groups (*P* > 0.05; Table 4).

**Table 4:** Comparison of postoperative imaging parameters between the two groups.

**Group**	**Group A** ***n* = 93**	**Group B** ***n* = 96**	***P***
Femoral prosthesis rotational alignment(°)	0.12 ± 1.3	−0.53 ± 1.3	0.005^*^
mFTA(°)	1.2 ± 0.6	1.0 ± 0.6	0.096

*Values are the means ± standard deviation*.

*mFTA, mechanical femoral axis–to–tibial axis angle*.

* *means there is significant difference between groups*.

#### KSS and VAS

The KSS and VAS scores were significantly improved after surgery in both groups. The mean KSS scores at 1, 3, 6, and 12 months after surgery were 73.3 ± 9.1, 80.3 ± 10.3, 84.7 ± 5.6 and 88.5 ± 5.1, respectively, in group A vs. 72.1 ± 9.0, 80.1 ± 11.3, 85.8 ± 7.9 and 87.1 ± 6.3, respectively, in group B; the differences between the two groups were not statistically significant (*P* > 0.05; Table 5). The VAS scores at 1, 3, 6, and 12 months after surgery were 3.8 ± 1.6, 1.5 ± 0.8, 1.3 ± 0.8 and 0.6 ± 0.6, respectively, in group A vs. 4.7 ± 1.5, 1.6 ± 1.1, 1.4 ± 0.8 and 0.7 ± 0.7, respectively, in group B; the differences between the two groups were not statistically significant at 3, 6, and 12 months, but mean VAS at 1 month after surgery was significantly better in group A than in group B (*P* = 0.008; Table 5).

**Table 5:** Comparison of postoperative KSS, VAS, ROM between the two groups.

	**Group**	**N**	**Preoperation**	**1 month**	**3 month**	**6 month**	**12 month**	***P***
KSS	Group A	48	41.6 ± 10.1	73.3 ± 9.1	80.3 ± 10.3	84.7 ± 5.6	88.5 ± 5.1	<0.001
	Group B	49	42.1 ± 9.0	72.1 ± 8.0	80.1 ± 11.3	85.8 ± 7.9	87.1 ± 6.3	<0.001
	*P*	-	0.806	0.505	0.916	0.461	0.264	-
VAS	Group A	48	5.0 ± 1.8	3.8 ± 1.6	1.5 ± 0.8	1.3 ± 0.8	0.6 ± 0.6	<0.001
	Group B	49	5.3 ± 1.6	4.7 ± 1.5	1.6 ± 1.1	1.4 ± 0.8	0.7 ± 0.7	<0.001
	*P*	-	0.419	0.008^*^	0.648	0.831	0.475	-
ROM	Group A	48	102.6 ± 9.2	109.7 ± 8.4	116.5 ± 9.6	118.7 ± 9.4	122.8 ± 8.6	<0.001
	Group B	49	103.7 ± 10.0	104.9 ± 8.4	116.1 ± 8.7	117.8 ± 8.5	121.3 ± 9.6	<0.001
	*P*	-	0.580	0.006^*^	0.831	0.641	0.417	-

*KSS, Knee Society Score; ROM, range of motion; VAS, visual analog scale*.

* *means there is significant difference between groups*.

#### Range of Motion

Mean range of motion at 1 month after surgery was significantly better in group A than in group B: 109.7° ± 8.4° vs. 104.9° ± 8.4° (*P* = 0.006; Table 5). However, the range of flexion at 3, 6, and 12 months after surgery was comparable between the groups (*P* > 0.05; Table 5).

## Discussion

The surgical approach and the degree of exposure influences soft tissue tension and thus affects the surgeon's judgment of soft tissue balance (12). This study showed that the joint gap at 90° of flexion was significantly smaller (0.4 mm) with the patella shifted laterally than with the patella reduced; the reduction of the lateral compartment space was also more obvious. This proves that subluxation of the patella intraoperatively will lead to underestimation of the joint gap at flexion, and that the soft tissue balance is more reliably estimated after the patella is reduced. Yoon et al. compared the changes in the extension and flexion gaps with the patella everted (15), subluxated, and reduced in patients undergoing traditional TKA. They found that the flexion gap was significantly smaller, and the valgus greater, when the patella was everted than when it was subluxated or reduced. The joint gap and varus angle were comparable with patellar subluxated and with patella reduced. The extension gap was not significantly affected by patella position. A cadaveric experiment by Gejo also indicated that eversion of the patella during traditional TKA might lead to underestimation of the flexion gap (16). It should be noted, of course, that the effect of patella position on soft tissue balance varies with the type of prosthesis used (17). All of our patients received the PS prosthesis.

As mentioned earlier, soft tissue tension is relatively high during MIS-TKA; subluxation of the patella further increases the tension. In fact, the patella subluxation will result in greater soft tissue tension than is seen with patella eversion in conventional TKA. The choice of the surgical approach (i.e., quadriceps-sparing (QS), midvastus, subvastus, or limited parapatellar approaches) influences the soft tissue tension. Oka et al. (18) compared the QS approach with the limited parapatellar approach and found that subluxation of the patella results in underestimation of the flexion gap in both groups of patients, though the effect was significantly more in patients receiving the QS approach. The varus angle changed significantly only in the QS group, probably due to the greater soft tissue tension generated when the patella was subluxated in the QS approach. Our study, in which the midvastus approach was used for all patients, also showed reduction of joint gap (0.4 mm) and varus angle (0.7 degree) after patellar subluxation. And most previous studies used the measured resection in operations while a modified gap balance technique was applied intraoperatively in ours. Keyser found that the effect of patellar subluxation on the flexion gap was mainly on the lateral gap in the MIS-TKA approach (19); this is consistent with the increase of the lateral gap and the increase of the varus angle after patellar reduction seen in in our study. According to Gejo, the flexion gap is also affected by patellar ligament tension, which may vary between patients (20). Therefore, to obtain accurate soft tissue balance, especially the gap balance in the flexion position, soft tissue balance must be judged with the patella reduced. According to Kamei, reduction in the bone mass effect of the distal femur after femoral osteotomy results in loosening of the patellar ligament and, consequently, an increase in the joint gap (21). To counteract this effect, we chose to do the measurement after the femoral prothesis was installed.

**Figure 4:**
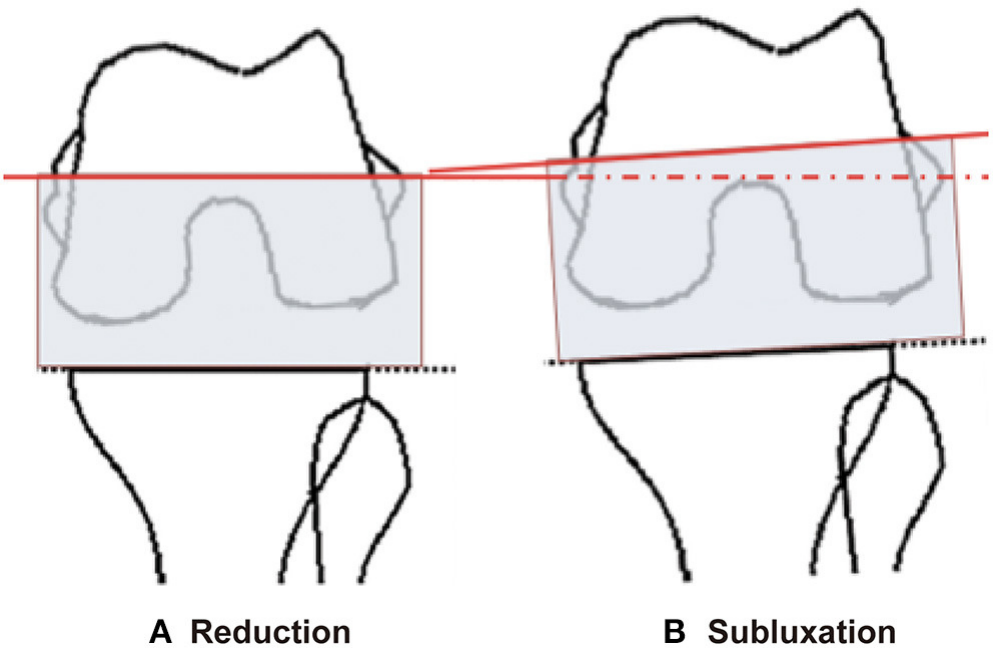
The tibial plateau was taken as the reference for the femoral posterior condylar cut using the gap-balance technique **(A)**. After subluxating the patellar **(B)**, varus angle of joint gap was smaller, so the inscribed line created by using the matching rectangular block parallel to the tibial plateau adds an internal rotation. This eventually caused the femoral prothesis to be placed internally rotated.

Recovery of the correct coronal limb alignment and femoral prosthesis rotation alignment are essential for good postoperative function. Poor coronal limb alignment can lead to residual deformities and accelerated wear of the prosthesis, while poor rotational alignment of the femoral prosthesis can cause imbalance of the flexion gap and poor patellar tracking, which will eventually affect knee function (22, 23). In this study we found that although there was no significant difference in the mFTA, the femoral prothesis was more internally rotated (0.65 degree) in group B patients. This was because when the patellar is subluxated at 90° flexion the reduction in gap is greater laterally than medially, which results in a reduction of the varus angle. As the tibial plateau was taken as the reference to perform osteotomy of the femoral posterior condylar with the gap-balance technique. After subluxating the patellar, varus angle of joint gap was smaller, so the scribe line recorded by using the matching rectangle parallel to tibial plateau was added an internal rotation and this eventually caused the installment of the femoral prothesis to be internally rotated (Figure 4). Therefore, we believe that in MIS-TKA, although patella position does not affect the reconstruction of the coronal limb alignment after surgery, the soft tissue balance achieved with the patella subluxated can lead to an increase in the internal rotation of the femoral prosthesis.

In this study, KSS, VAS, and range of flexion showed significant improvement after surgery. The improvement in the ROM and VAS were better in group A at the first follow-up visit; this may be related to better patellar tracking and physiological balance of soft tissue in this group. No significant difference in KSS was observed between the two groups. Many factors other than the surgical technique affect patient satisfaction; these may include the patient's medical history, pain threshold, mental status, and expectations (24–26). All of these also deserve the attention of the surgical team before and after surgery.

There are some limitations in this study. First, patients with valgus were excluded and most patients were women; these factors may have biased the results. Second, previous literature indicates that the application of a tourniquet may affect the extensor mechanism; this could theoretically affect the balance of the intraoperative gap, we could not avoid the use of tourniquets in this study. Finally, the limited number of patient and incomparable gender ratio might have a bias to the results. And the follow-up time was short, so no conclusions can be drawn on prosthetic wear and survival rates.

## Conclusions

The impact of the patellar position on the flexion gap should be considered when assessing soft tissue balance during MIS-TKA. As far as possible, soft tissue balance must be achieved with the patellar reduced; otherwise, the femoral prosthesis may be installed relatively internally and, after patella reduction, the flexion gap and the varus would increase.

## Data Availability Statement

The original contributions presented in the study are included in the article/supplementary material, further inquiries can be directed to the corresponding authors.

## Ethics Statement

The studies involving human participants were reviewed and approved by Ethics Committee of Shanghai Tenth People's Hospital. The patients/participants provided their written informed consent to participate in this study.

## Author Contributions

KL designed the study and wrote the article. All authors contributed to the article and approved the submitted version.

## Funding

This work was supported by the National Natural Science Foundation of China (NSFC), grant number (81874125).

## Conflict of Interest

The authors declare that the research was conducted in the absence of any commercial or financial relationships that could be construed as a potential conflict of interest.

## Publisher's Note

All claims expressed in this article are solely those of the authors and do not necessarily represent those of their affiliated organizations, or those of the publisher, the editors and the reviewers. Any product that may be evaluated in this article, or claim that may be made by its manufacturer, is not guaranteed or endorsed by the publisher.
